# Supplementary material for: Computational reconstruction of mental representations using human behavior
Source: Nat Commun. 2024 May 17;15:4183. doi: 10.1038/s41467-024-48114-6 (PMC11101448; doi:10.1038/s41467-024-48114-6)
Supplement: Supplementary file 1 — Supplementary Information [file 41467_2024_48114_MOESM1_ESM.pdf]

Computational reconstruction of mental representations using human behavior

Supplementary Information

Laurent Caplette[1,*] & Nicholas B. Turk-Browne[1,2]

[1]Department of Psychology, Yale University, New Haven, CT, U.S.A.

[2]Wu Tsai Institute, Yale University, New Haven, CT, U.S.A.

*Corresponding author

Email: laurent.caplette@yale.edu

Address: Department of Psychology, Yale University, 100 College Street, New Haven, CT, 06510, U.S.A.

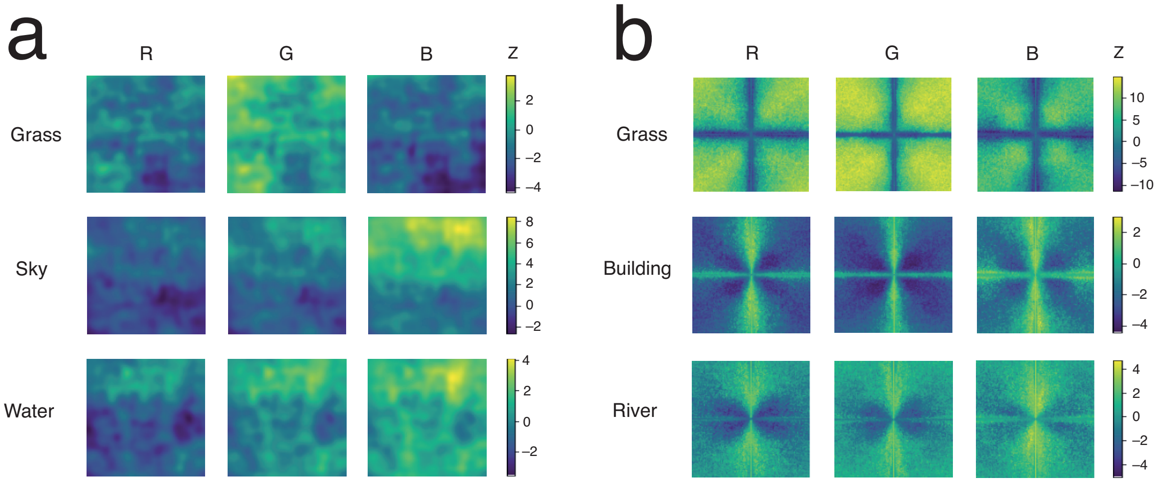

Figure S1. Analyses using other feature spaces. **A)** Analysis using pixel space. An analysis similar to the main analysis was performed but using the pixel values of the stimuli as features (instead of the CNN features). Additional smoothing was performed to increase signal-to-noise ratio. The regression coefficients for each pixel and each color channel are displayed. Lighter colors (higher z-scores) indicate that these image regions and color channels correlate more to the semantic features of the concept. **B)** Analysis using the Fourier power spectrum. Same as (A) but using the values of the Fourier power spectrum at each orientation and spatial frequency (SF; up to 64 cycles per image). Here, no smoothing was applied but 10 principal components were included to retain 99.9% of the variance. The regression coefficients for each SF, orientation and color channel are displayed. Lower SFs are at the center. Lighter colors (higher z-scores) indicate that these SFs and orientations correlate more with the semantic features of the concept.

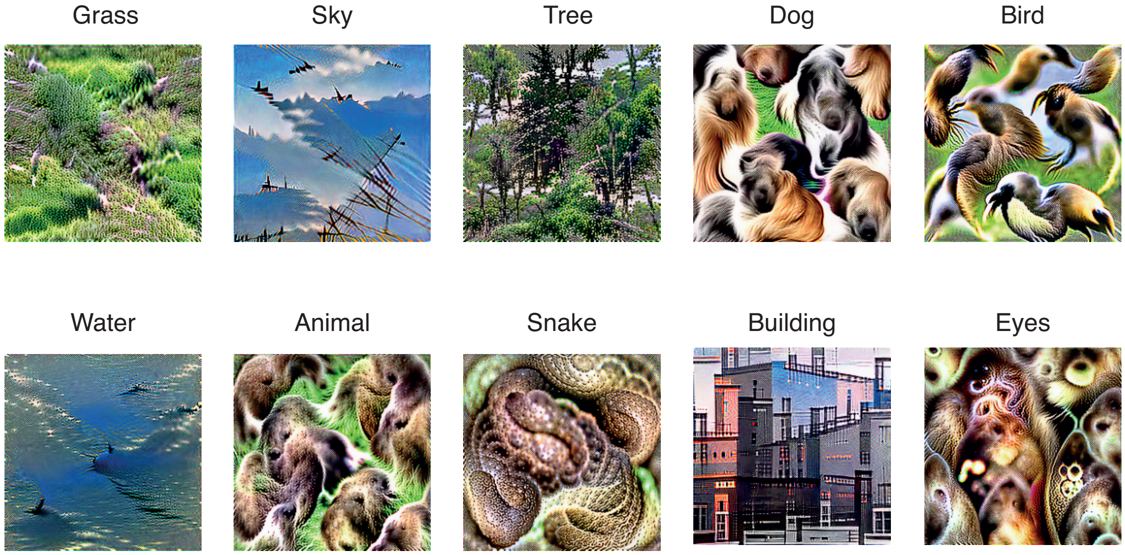

Figure S2. Reconstructions of the representations of the 10 most-named concepts using the preregistered *word2vec* word embedding.

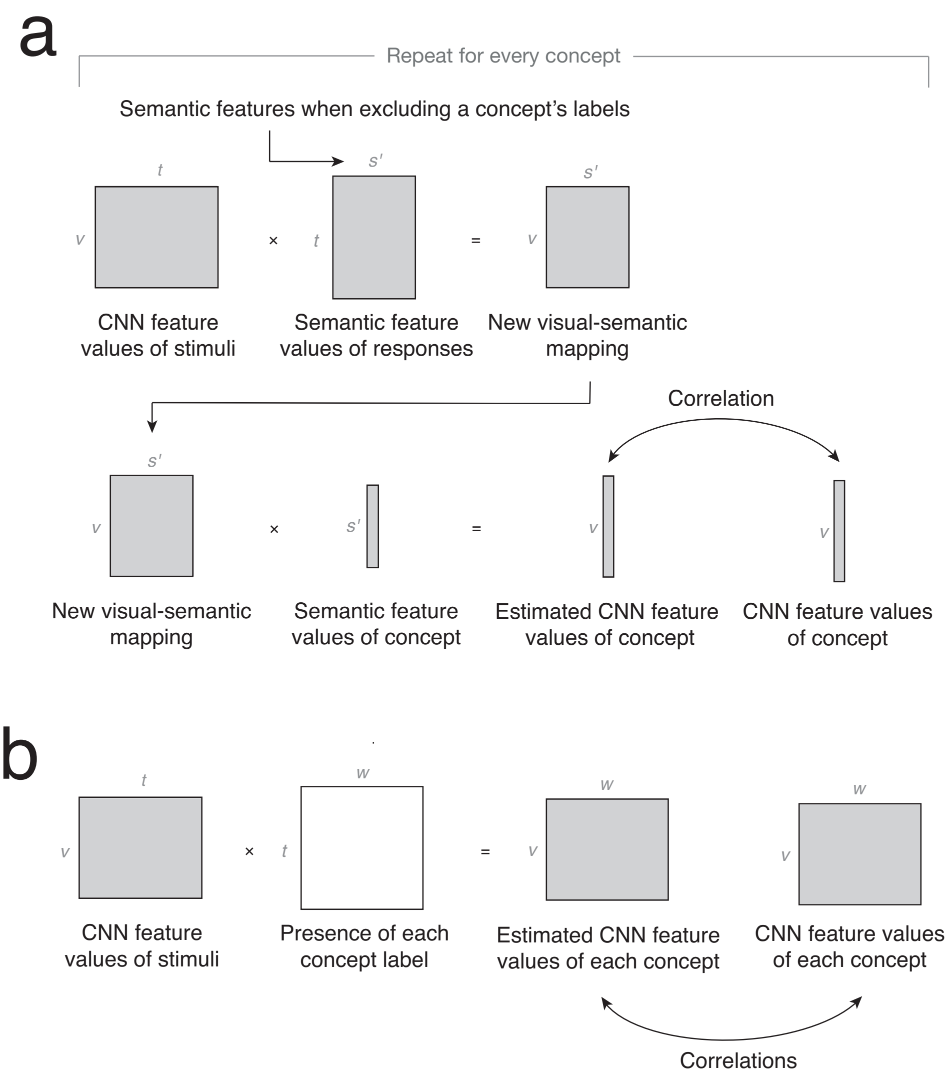

Figure S3. Investigating the effect of the semantic embedding. See Figure 5, panels d and e. Gray squares represent matrices made of real numbers, white squares represent binary matrices of Booleans. **A)** Analysis while excluding all responses containing a concept's label. The following procedure was applied for each analyzed concept: compute the semantic feature values associated with every trial, but excluding the responses that are the concept's label; perform an outer product of this matrix with the matrix of CNN feature values as in the main analysis (the result is a new visual-semantic mapping); multiply this matrix with the semantic feature values of the concept; correlate the resulting CNN feature values with the CNN feature values obtained in the main analysis. v = CNN features; t = trials; s′ = new semantic features. **B)** Analysis without a semantic embedding. The semantic embedding was replaced with a binary matrix of whether each word (among the 369 words named at least 10 times) was answered or not on each trial. This matrix was multiplied by the CNN feature values of all stimuli to obtain the CNN feature values associated with all words named more than 10 times. These CNN feature values were finally correlated with the CNN feature values obtained in the main analysis. v = CNN features; t = trials; w = words.

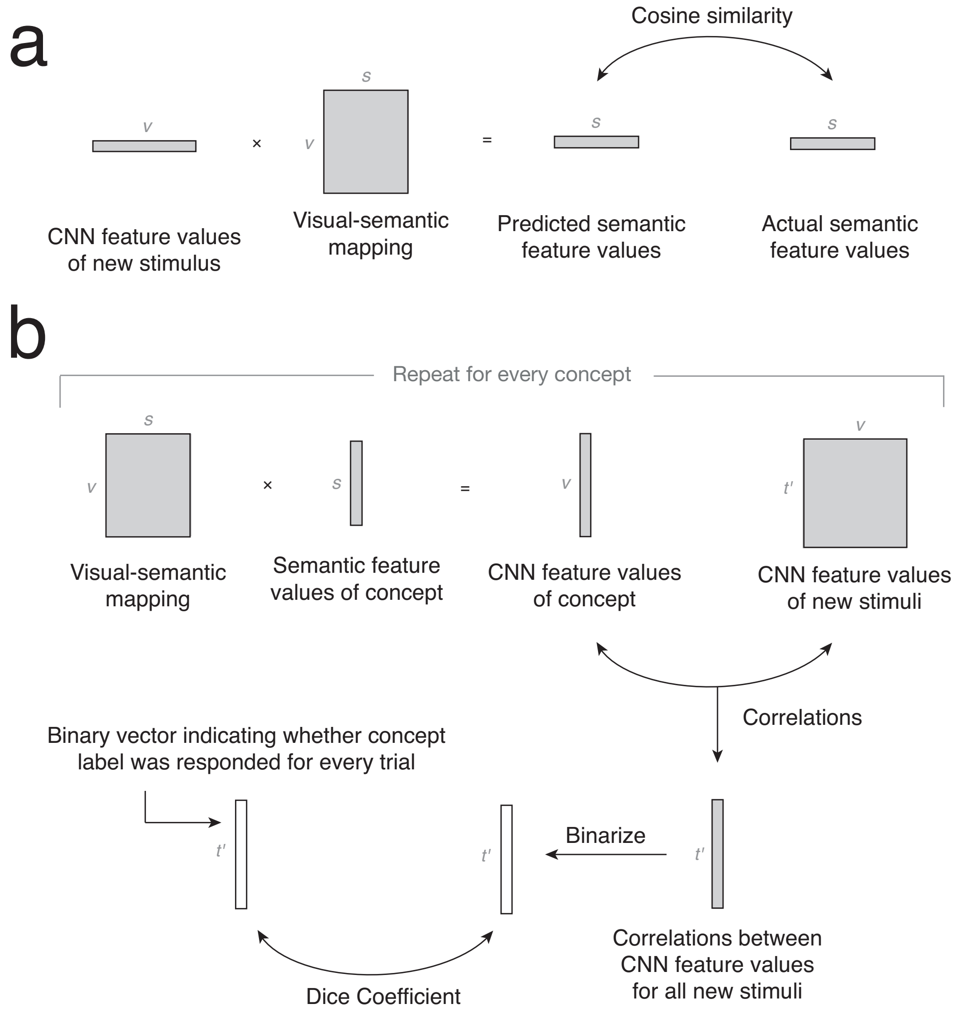

Figure S4. Prediction analyses. See Figure 6, panels a and b. Gray squares represent matrices made of real numbers, white squares represent binary matrices of Booleans. **A)** Prediction of the semantic content of new stimuli. To predict the semantic feature values of a new stimulus, we multiplied its CNN feature values by the visual-semantic mapping. This resulted in predicted semantic feature values for that stimulus. We compared these predicted semantic feature values to its actual semantic feature values by computing their cosine similarity. v = CNN features; s = semantic features. **B)** Prediction of the stimuli classified as a given concept. The following procedure was applied for each analyzed concept: the CNN feature values of the concept were computed by multiplying the visual-semantic mapping and the concept's semantic feature values; these CNN feature values were correlated to the CNN feature values of a set of new stimuli; the resulting vector of correlation values for each stimulus was thresholded so that the number of stimuli above threshold was equal to the number of stimuli containing that concept's label as an answer; finally, the match of these binary vectors was quantified with a Dice coefficient. v = CNN features; s = semantic features; t′ = new trials.

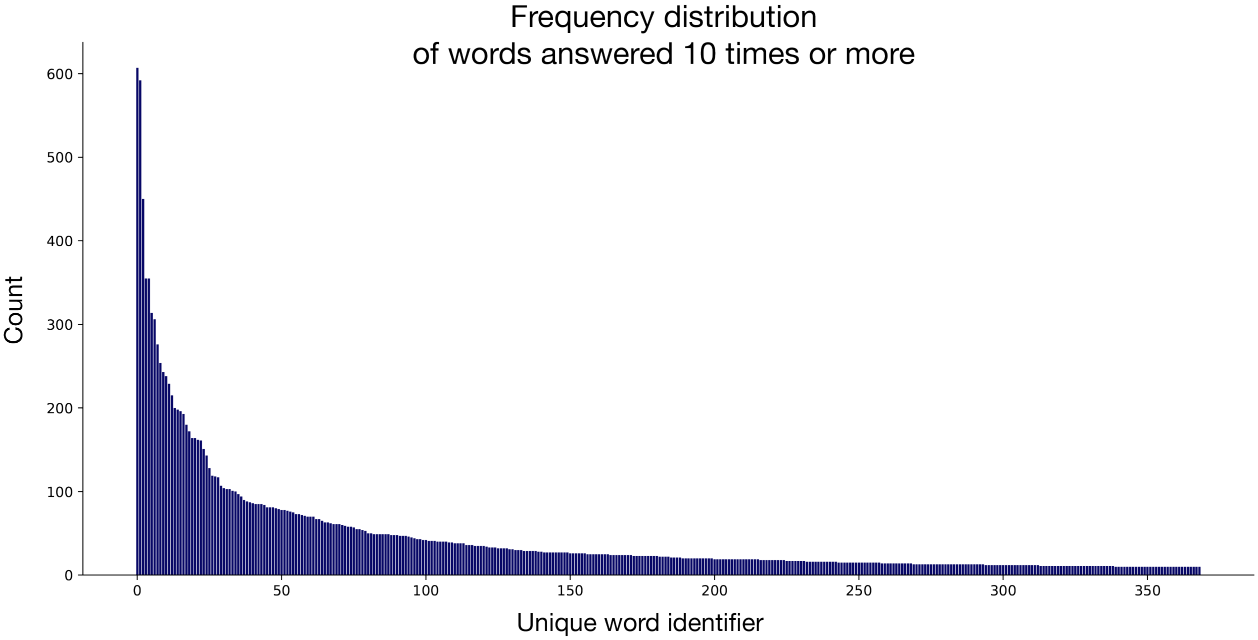

Figure S5. Frequency distribution of all words answered at least 10 times in the main experiment, ordered from most answered to least answered (see also Table S1).

1. grass
2. sky
3. tree
4. dog
5. bird
6. water
7. animal
8. snake
9. building
10. eyes
11. car
12. person
13. eye
14. house
15. wall
16. window
17. face
18. cat
19. fish
20. chair
21. room
22. forest
23. sand
24. trees
25. monkey
26. flower
27. table
28. door
29. cloud
30. field
31. owl
32. snow
33. leaf
34. city
35. sea
36. couch
37. plant
38. desert
39. fire
40. light
41. mountain
42. bear
43. spider
44. stairs
45. bed
46. horse
47. plants
48. lake
49. fence
50. turtle
51. bridge
52. road
53. carpet
54. boat
55. ocean
56. elephant
57. insect
58. frog
59. glass
60. smoke
61. man
62. lamp
63. hand
64. fur
65. flowers
66. seat
67. street
68. floor
69. sofa
70. people
71. dirt
72. lights
73. night
74. butterfly
75. beach
76. rabbit
77. clouds
78. train
79. lizard
80. computer
81. kitchen
82. sunset
83. mouse
84. wood
85. space
86. sun
87. tower
88. mirror
89. squirrel
90. ice
91. cactus
92. river
93. cow
94. machine
95. leaves
96. skin
97. wolf
98. camera
99. snail
100. lion
101. farm
102. green
103. nose
104. ship
105. duck
106. gorilla
107. painting
108. blanket
109. hair
110. bathroom
111. plane
112. worm
113. robot
114. garden
115. penguin
116. eagle
117. feather
118. head
119. bag
120. woods
121. woman
122. clothes
123. explosion
124. sheep
125. hallway
126. rock
127. goat
128. pool
129. bush
130. bug
131. fox
132. rat
133. sign
134. outdoor
135. park
136. television
137. store
138. ball
139. feathers
140. mushroom
141. screen
142. skull
143. radio
144. chicken
145. metal
146. branch
147. wheat
148. whale
149. greenhouse
150. shell
151. phone
152. money
153. hamster
154. bee
155. leather
156. ground
157. reptile
158. stone
159. furniture
160. ceiling
161. shoe
162. map
163. curtain
164. fly
165. ghost
166. plastic
167. vegetation
168. shadow
169. cup
170. gun
171. parrot
172. peacock
173. pig
174. baby
175. bottle
176. octopus
177. egg
178. skyscraper
179. church
180. girl
181. coral
182. box
183. toilet
184. sloth
185. white
186. tiger
187. teeth
188. fabric
189. palm
190. deer
191. hat
192. buildings
193. desk
194. keyboard
195. airplane
196. roof
197. mask
198. book
199. pyramid
200. leg
201. dragon
202. mouth
203. black
204. brick
205. paper
206. bedroom
207. pillow
208. hole
209. scale
210. fridge
211. moth
212. tv
213. scales
214. alien
215. net
216. outside

217. human
218. bat
219. fruit
220. waterfall
221. statue
222. swamp
223. camel
224. arm
225. giraffe
226. office
227. dessert
228. finger
229. crocodile
230. skunk
231. seaweed
232. jungle
233. factory
234. brain
235. bunny
236. pole
237. bench
238. sink
239. bricks
240. storm
241. candle
242. dinosaur
243. guitar
244. earth
245. landscape
246. animals
247. sealant
248. pipe
249. button
250. dark
251. elevator
252. ear
253. moss
254. turkey
255. bowl
256. wheel
257. rockslide
258. walls
259. rocket
260. shark
261. wool
262. panda
263. cinema
264. aquarium
265. cliff
266. helmet
267. truck
268. pond
269. algae
270. doorway
271. monitor
272. shop
273. wing
274. wave
275. microwave
276. jewelry
277. towel
278. bus
279. tunnel
280. koala
281. restaurant
282. bacteria
283. sidewalk
284. cushion
285. branches
286. swan
287. birds
288. wire
289. antenna
290. seats
291. nature
292. telephone
293. hawk
294. pet
295. air
296. monster
297. shelf
298. flamingo
299. dolphin
300. speaker
301. spaceship
302. ostrich
303. entrance
304. bushes
305. belt
306. color
307. paw
308. skyline
309. gold
310. blood
311. oven
312. food
313. wind
314. volcano
315. snails
316. body
317. rain
318. windowsill
319. leopard
320. web
321. rose
322. clock
323. highway
324. board
325. fireplace
326. traffic
327. tail
328. tongue
329. knife
330. darkroom
331. railing
332. coffee
333. station
334. vase
335. game
336. rug
337. chocolate
338. dashboard
339. brown
340. iguana
341. tornado
342. mud
343. lounge
344. basket
345. atm
346. airport
347. soap
348. land
349. legs
350. mountainside
351. neon
352. jacket
353. shirt
354. lighthouse
355. gravel
356. hill
357. gas
358. puppy
359. stadium
360. cake
361. barn
362. crops
363. foot
364. living-room
365. electronics
366. slug
367. caterpillar
368. apartment
369. abyss

Table S1. List of all words answered at least 10 times in the main experiment, ordered from most answered to least answered (see also Figure S5).
