# Supplementary material for: Computational reconstruction of mental representations using human behavior
Source: Nat Commun. 2024 May 17;15:4183. doi: 10.1038/s41467-024-48114-6 (PMC11101448; doi:10.1038/s41467-024-48114-6)
Supplement: Supplementary file 3 — Reporting Summary [file 41467_2024_48114_MOESM3_ESM.pdf]

nature portfolio

Corresponding author(s): Laurent Caplette

Last updated by author(s): Apr 1, 2024

Reporting Summary

Nature Portfolio wishes to improve the reproducibility of the work that we publish. This form provides structure for consistency and transparency in reporting. For further information on Nature Portfolio policies, see our Editorial Policies and the Editorial Policy Checklist.

Statistics

For all statistical analyses, confirm that the following items are present in the figure legend, table legend, main text, or Methods section.

n/a	Confirmed	
☐	☒	The exact sample size (*n*) for each experimental group/condition, given as a discrete number and unit of measurement
☐	☒	A statement on whether measurements were taken from distinct samples or whether the same sample was measured repeatedly
☐	☒	The statistical test(s) used AND whether they are one- or two-sided<br>*Only common tests should be described solely by name; describe more complex techniques in the Methods section.*
☒	☐	A description of all covariates tested
☐	☒	A description of any assumptions or corrections, such as tests of normality and adjustment for multiple comparisons
☐	☒	A full description of the statistical parameters including central tendency (e.g. means) or other basic estimates (e.g. regression coefficient) AND variation (e.g. standard deviation) or associated estimates of uncertainty (e.g. confidence intervals)
☐	☒	For null hypothesis testing, the test statistic (e.g. *F*, *t*, *r*) with confidence intervals, effect sizes, degrees of freedom and *P* value noted<br>*Give P values as exact values whenever suitable.*
☒	☐	For Bayesian analysis, information on the choice of priors and Markov chain Monte Carlo settings
☒	☐	For hierarchical and complex designs, identification of the appropriate level for tests and full reporting of outcomes
☐	☒	Estimates of effect sizes (e.g. Cohen's *d*, Pearson's *r*), indicating how they were calculated

Our web collection on statistics for biologists contains articles on many of the points above.

Software and code

Policy information about availability of computer code

Data collection	PsychoPy/PsychoJS (2020.1; www.psychopy.org); Pavlovia website (pavlovia.org); Predictionary (github.com/asterics/predictionary); custom code (github.com/laurentcaplette/Representation-reconstruction).
Data analysis	Robustness package (1.2.1; git.io/robust-reps); NumPy (1.21.5; numpy.org); Scikit-Learn (0.23.2; scikit-learn.org); Scipy (1.7.3; scipy.org); PyTorch (1.5; pytorch.org); TorchVision (0.6.0; pytorch.org); NLTK (3.7; www.nltk.org); SymSpell (6.7.0; github.com/wolfgarbe/SymSpell; custom code (https://github.com/laurentcaplette/Representation-reconstruction).

For manuscripts utilizing custom algorithms or software that are central to the research but not yet described in published literature, software must be made available to editors and reviewers. We strongly encourage code deposition in a community repository (e.g. GitHub). See the Nature Portfolio guidelines for submitting code & software for further information.

Data

Policy information about availability of data

All manuscripts must include a data availability statement. This statement should provide the following information, where applicable:

- Accession codes, unique identifiers, or web links for publicly available datasets
- A description of any restrictions on data availability
- For clinical datasets or third party data, please ensure that the statement adheres to our policy

All raw and preprocessed data generated and analyzed during this study are available at https://osf.io/mp3s6/ (DOI: 10.17605/OSF.IO/MP3S6). The following publicly available data were also used in the study: Behavioral dataset on semantic word arrangement(https://osf.io/um3qg/); Visual Genome dataset (https://

homes.cs.washington.edu/~ranjay/visualgenome/index.html); GloVe word embedding (https://nlp.stanford.edu/projects/glove/); pretrained adversarially robust ResNet-50 (git.io/robust-reps)); ImageNet Large-Scale Visual Recognition Challenge (ILSVRC) 2012 dataset (https://www.image-net.org/challenges/LSVRC/2012/).

Research involving human participants, their data, or biological material

Policy information about studies with human participants or human data. See also policy information about sex, gender (identity/presentation), and sexual orientation and race, ethnicity and racism.

Reporting on sex and gender	Sex and gender were not considered in study design and information was not collected (except for in-person sample). They were not considered to be relevant to the questions under investigation.
Reporting on race, ethnicity, or other socially relevant groupings	Race, ethnicity, or other socially relevant groupings were not used in the manuscript.
Population characteristics	Healthy adults aged 18-35 with normal or corrected-to-normal vision.
Recruitment	Recruited on the Prolific platform and on the Yale University campus. Selection biases are possible because these samples are not randomly drawn from the population. This could have affected how our results are representative of the overall population.
Ethics oversight	Yale University Institutional Review Board

Note that full information on the approval of the study protocol must also be provided in the manuscript.

Field-specific reporting

Please select the one below that is the best fit for your research. If you are not sure, read the appropriate sections before making your selection.

☐ Life sciences ☒ Behavioural & social sciences ☐ Ecological, evolutionary & environmental sciences

For a reference copy of the document with all sections, see nature.com/documents/nr-reporting-summary-flat.pdf

Behavioural & social sciences study design

All studies must disclose on these points even when the disclosure is negative.

Study description	Online experimental studies with human subjects; quantitative data.
Research sample	Samples are from the Prolific participant database and the Yale University Department of Psychology. Samples are not strictly representative. They were chosen because they allowed us to collect large amounts of high-quality data. The participants are all healthy adults aged between 18-35 with normal or corrected-to-normal vision. The sex of the participants in the online samples is unknown; in the in-person sample, there were 5 women and 3 men.
Sampling strategy	Samples of convenience. Sample size was preregistered; it was chosen based on previous literature and pilot study.
Data collection	Data collection was online. Participants had to use their keyboards. There were no experimental conditions per se. The researcher was not blind to the study hypotheses.
Timing	Main study: October 15-16, 2020 + November 5, 2020 (the gap is explained by the fact that we had to collect additional data after the exclusion of participants based on their data; this procedure and the exclusion criteria were preregistered). Validation study #1: June 30, 2021 to July 1st, 2021. Individual representations study: January 21, 2021 to March 25, 2021. Validation study #2: June 29-30, 2022. Validation study #3: April 26, 2023.
Data exclusions	17 exclusions in main study based on number of non-concrete words; 2 exclusions in validation study #1 based on mean accuracy; 6 exclusions in validation study #3 based on validity of responses. Exclusion criteria were pre-established.
Non-participation	Only the individual representations study required multiple sessions and no participant dropped out.
Randomization	Participants were not allocated into experimental groups.

Reporting for specific materials, systems and methods

We require information from authors about some types of materials, experimental systems and methods used in many studies. Here, indicate whether each material, system or method listed is relevant to your study. If you are not sure if a list item applies to your research, read the appropriate section before selecting a response.

Materials & experimental systems

n/a	Involved in the study
☒	☐ Antibodies
☒	☐ Eukaryotic cell lines
☒	☐ Palaeontology and archaeology
☒	☐ Animals and other organisms
☒	☐ Clinical data
☒	☐ Dual use research of concern
☒	☐ Plants

Methods

n/a	Involved in the study
☒	☐ ChIP-seq
☒	☐ Flow cytometry
☒	☐ MRI-based neuroimaging

Plants

Seed stocks	n/a
Novel plant genotypes	n/a
Authentication	n/a
